# 1137. The Value of Invasive Meningococcal Disease Combination Vaccine – a Qualitative Study of Adolescents and Parents/Caregivers’ Preferences in the US

**DOI:** 10.1093/ofid/ofad500.978

**Published:** 2023-11-27

**Authors:** Shahina Begum, Eliazar Sabater Cabrera, Linda Hortobagyi, Twinkle Khera, Selene Camargo Correa, Laurie Batchelder, Zeki Kocaata

**Affiliations:** GSK, London, England, United Kingdom; GSK, London, England, United Kingdom; GSK, London, England, United Kingdom; IQVIA, Bangalore, Karnataka, India; IQVIA, Bangalore, Karnataka, India; IQVIA, Bangalore, Karnataka, India; GSK, London, England, United Kingdom

## Abstract

**Background:**

MenACWY and MenB are commonly used vaccines to prevent invasive meningococcal disease (IMD), targeting serogroups A, B, C, W, Y. MenABCWY combination vaccines are under development and could provide increased vaccine coverage of serogroups. This qualitative study aimed to identify concepts affecting preferences in adolescents (Ado) and parents/caregivers (P/C) decision making towards combination vaccine in the US.

**Methods:**

Two focus group discussions (FGD), 90-minutes with Ado (16-23 years) and P/C of adolescents (16-18 years) were conducted (Table 1). Guides were developed based on a targeted literature review to investigate preferences for potential features of a combination vaccine. Participants were presented with IMD and vaccines information. Important/least important factors for decision-making were transcribed in response to open-ended/probes questions. FGDs were coded to apply thematic assessment. Results were synthesized separately by moderator-probed and spontaneously mentioned themes. Percentages were calculated on participant numbers contributing to a theme.

**Figure:**
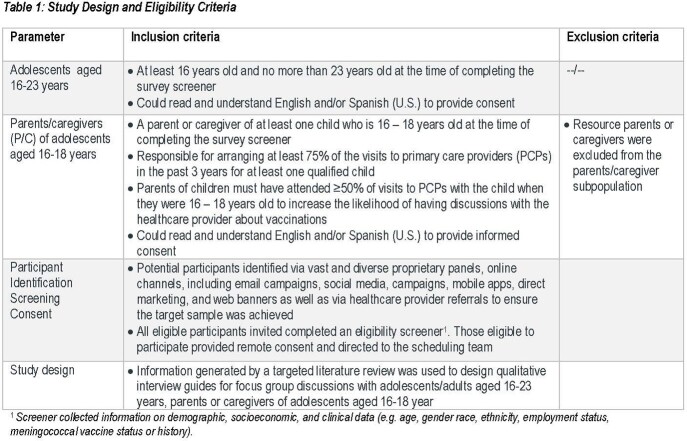

**Results:**

Thirteen participants were included in FGDs (6 Ado, 7 P/C, 57% P/C with college or lower degree, Table 2). Ado preferred a combination vaccine which provided time saving (100%) and convenience (83%) by reducing the number of injections in the immunization series (100%) and number of visits (100%). P/C considered injection site discomfort (71%) as an important decision factor for a combination vaccine, however Ado considered this as least important (100%). Both groups considered impact on healthcare system and environment as least important for a combination vaccine (55%) (Table 3). Cross-protection against other infectious disease (55%, probed) and spontaneous themes (e.g. duration of protection, effectiveness, side effects and dosing interval) emerged (Table 4). These concepts were considered relevant for combination vaccine decision-making, although could be applicable to IMD vaccination more generally.

**Figure:**
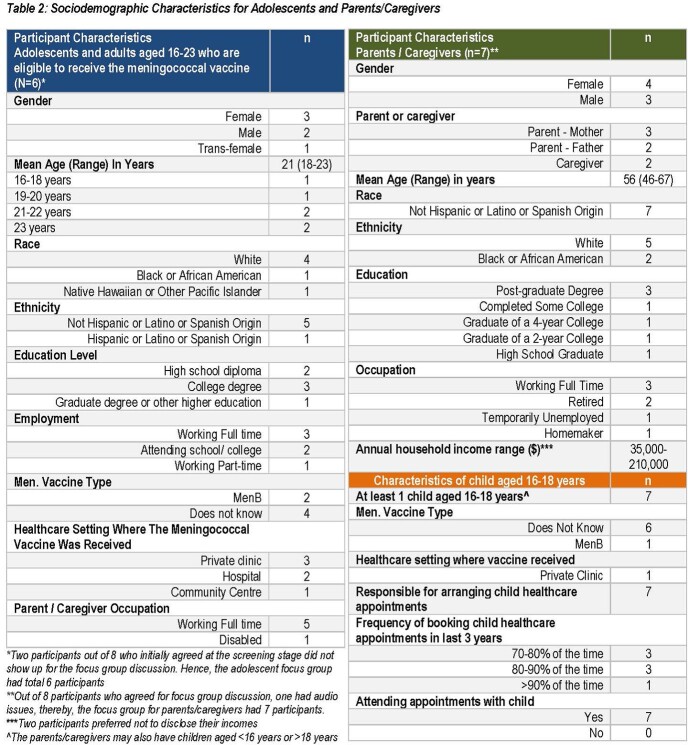

**Figure:**
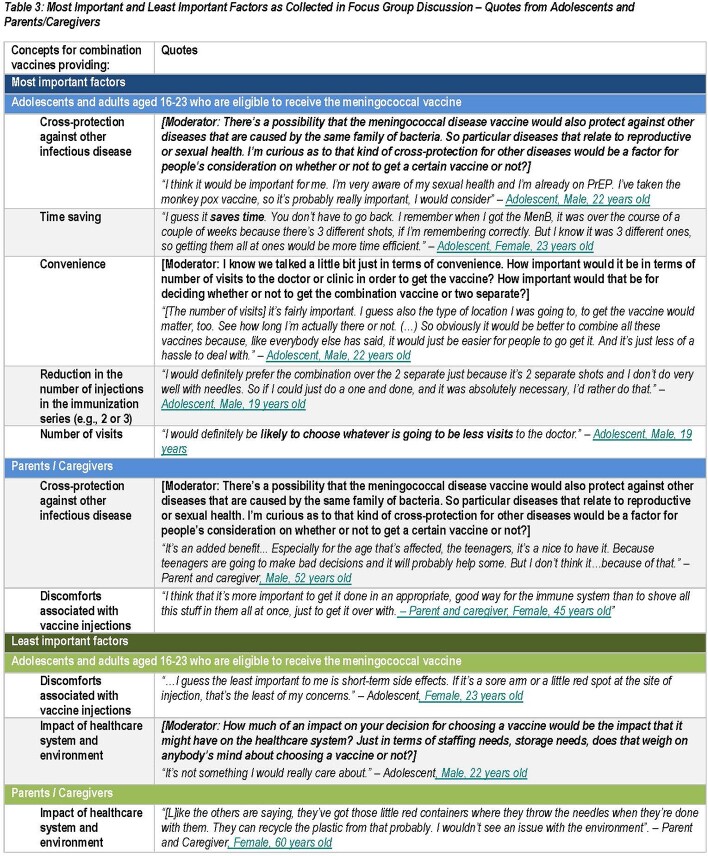

**Figure:**
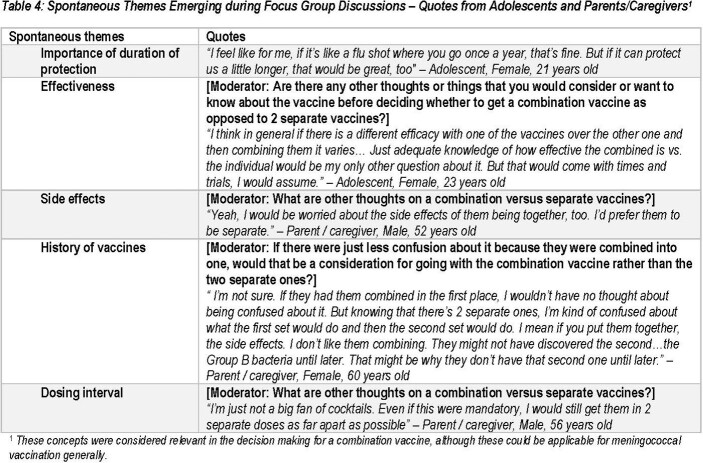

**Conclusion:**

The findings suggest vaccine-receivers preferred a combination vaccine covering serogroups A, B, C, W, Y, with simplified schedules (e.g. fewer visits and injections) and potential cross-protection against other infectious diseases.

**Disclosures:**

**Shahina Begum**, GSK: Employee **Eliazar Sabater Cabrera, PhD**, GSK: Employee|GSK: Stocks/Bonds **Linda Hortobagyi, MSc**, GSK: Contractor **Twinkle Khera, Mtech**, IQVIA: Advisor/Consultant **Selene Camargo Correa, PhD**, IQVIA: Advisor/Consultant **Laurie Batchelder, PhD**, IQVIA: Advisor/Consultant **Zeki Kocaata, PhD**, GSK: Stocks/Bonds

